# Gait Improvements After Peroneal or Tibial Nerve Transfer in Patients with Foot Drop: A Retrospective Study

**Published:** 2017-09-29

**Authors:** Rahul K. Nath, Chandra Somasundaram

**Affiliations:** Texas Nerve and Paralysis Institute, Houston

**Keywords:** foot drop, peroneal nerve injury, nerve transfer, dorsiflexion, antigravity

## Abstract

**Background:** Injury to the common peroneal nerve disrupts the motor control pathway to ankle dorsiflexors and evertors, as well as toe extensors, resulting in pathological gait and foot drop. Direct external compression on the fibular head is the most frequent cause of peroneal nerve impairment and has poor prognosis. **Methods and Patients:** Here, we report the surgical outcome of 21 patients with foot drop (9 males and 12 females) who underwent nerve transfer procedure of either the superficial peroneal nerve or the tibial nerve fascicles to the motor branch of the tibialis anterior and to the deep peroneal nerve. They had at least 6 months postoperative follow-up (mean = 17; range, 6-32 months). **Results:** Among 21 patients who had no ankle dorsiflexion (BMRC 0/5) preoperatively, 9 patients had successful restoration of ankle dorsiflexion (BMRC 4 to 4+/5), 7 patients had BMRC 2 to 3+/5, and 4 patients had no or poor restoration of dorsiflexion (BMRC 0 to 1+/5) but achieved good ankle eversion (BMRC 3 to 4+/5). Overall statistically significant clinical improvement of ankle dorsiflexion and eversion from preoperative BMRC grade 2.6 ± 0.5 to postoperative BMRC grade 3.6 ± 0.7 (*P* = .0000004) was achieved. **Conclusion:** Overall statistically significant clinical improvement of ankle dorsiflexion and eversion was achieved in 80% of our study patients. Most of these patients gained antigravity and were able to walk with minimal steppage gait. In the other 4 patients (20%), there was good improvement in ankle eversion but poor or no ankle dorsiflexion.

Injury to the common peroneal nerve causes antigravity weakness to the tibialis anterior and peroneus muscles (ankle dorsiflexors and evertors, as well as toe extensors), results in slapping or tripping of foot, decreased mobility, and reduced functions, and affects overall quality of the life.

This is the most frequent entrapment in lower extremity and about 15% of all mononeuropathies in adults and has a worse prognosis than those of the upper extremities.^1^^-^4 Foot drop resulting from peroneal nerve injury is caused by both external and internal factors. However, direct external compression on the fibular head is the most common cause of this impairment.

Peroneal nerve damage can occur due to accident, gunshot wound, sciatic nerve tumor, radiculopathy, and hip and knee replacement surgery in numerous pathologies such as stroke, spinal cord injury, multiple sclerosis, and cerebral palsy. Generally, these impairments are complex to manage successfully. The treatment strategies vary according to the nature and level of the nerve damage and planned depending on the underlying pathology.

Several management techniques, including surgical (nerve transfer/grafting, and tendon transfers) and nonsurgical treatments (ankle-foot orthosis [AFO]), have been adopted to improve the failed gait and dropped foot. Nerve stimulators are used^5^^,^^6^ if the patient has the intact functioning peroneal nerve. If there is no evidence of functional recovery, surgical exploration is necessary and performed at 3 to 7 months from injury.^7^^,^^8^ Surgical procedure and time of intervention also depend on the type and degree of injury. Laceration or open wounds of the peroneal nerve undergo immediate surgical exploration.^9^ Other lesions are generally followed clinically and examined electromyographically.

Nerve grafting has been reported unsuccessful if the grafts are more than 6 cm in length,^8^^,^^10^^,^^11^ as this is the case in most traumatic longitudinal nerve traction injuries. Tibial tendon transfer has been shown to improve patients’ mobility/ambulation without assistive devices. However, tendon transfer has been reported to provide only weaker dorsiflexion and gait.^12^ We report here the results of a retrospective analysis of surgically managed 21 patients who had knee injuries and foot drop.

## METHODS AND PATIENTS

This is a retrospective study of a series of 21 consecutive patients (12 females and 8 males) with knee injuries and foot drop. Patients in our present study underwent peroneal or tibial nerve transfer with us between 2006 and 2015. The lead author (R.K.N.)^13^ performed decompression, internal neurolysis, and external neuroplasty of the affected peroneal nerve in all patients and the tibial nerve in some patients. Furthermore, we transferred tibial or superficial peroneal nerve fascicles to the motor branch of the tibialis anterior and deep peroneal nerve.^13^ Excision of neuroma of the deep peroneal nerve was also done in 1 patient. Intraoperative monitoring of the peroneal nerve was performed on the affected leg in all patients in this study.

Mean follow-up after the operation was 19 months (range, 6–50 months). The mean age was 44 years (range, 17–77 years). For all patients, the preoperative clinical examination and electromyographic evaluation were performed. Nerve conduction and clinical function results showed injury to the peroneal nerve in all patients. Preoperative duration of injury and foot drop (1–17 months) reported by these patients was recorded for all patients except one. Further patient demographics, including age, gender, mechanism, and extent of the injury, are provided in Table 1.

The lead author (R.K.N.) clinically evaluated ankle eversion, inversion, dorsiflexion, and plantar flexion using Modified British Medical Research Council Motor Scale (BMRC) for muscle strength preoperatively and at least 6 months after surgery.

This was a retrospective study of patient charts, which exempted it from the need for institutional review board approval in the United States. Patients were treated ethically in compliance with the Helsinki declaration. Documented informed consent was obtained for all patients.

### Statistical analysis

Statistical analysis was performed using Analyse-It plugin (Leeds, United Kingdom) for Microsoft Excel 2003 software. A *P* value of less than .05 was considered as statistically significant.

## RESULTS

Our patients in this study had peroneal nerve injuries and foot drop resulting from mostly lumbar surgical procedures, accidents, and gunshot wounds. The outcomes of the injured foot movements for each of these 21 patients are shown separately in Table 1. Foot movements from tibial nerve innervations are toe flexion, plantar flexion, and ankle inversion. These were not greatly affected and therefore no significant changes after the surgery. Foot movements from peroneal nerve innervations are ankle dorsiflexion and eversion. These foot functions were severely damaged to mean BMRC 1.1 as compared with the normal value of BMRC 5.0. Overall statistically significant clinical improvement of ankle dorsiflexion, eversion, and plantar flexion from preoperative BMRC grade 2.6 ± 0.5 to postoperative BMRC grade 3.6 ± 0.7 (*P* = .0000004) was achieved (Table 2).

**Figure 1:**
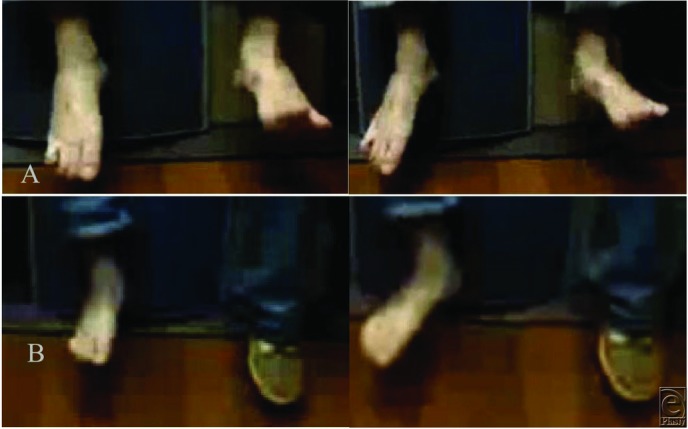

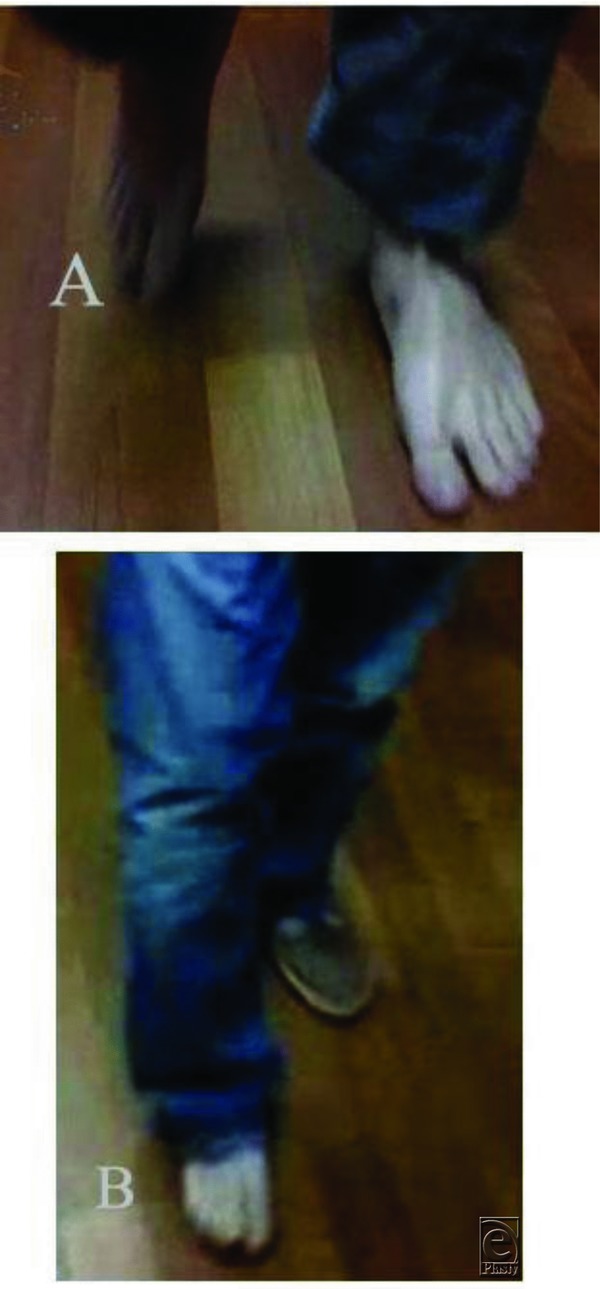
(a) A 24-year-old male patient with right peroneal nerve injury and foot drop resulting from anterior cruciate ligament repair (per patient). Upper panel (A), right foot: The patient was unable to dorsiflex the ankle (BMRC 0/5) before surgery. Lower panel (B): Significant improvement in ankle dorsiflexion (BMRC 4/5), toe extension, and eversion after surgery (peroneal nerve decompression, microneurolysis, neuroplasty, and nerve transfer). (b) Upper panel (A): Steppage gait before surgery. Lower panel (B): The patient was able to walk without slapping or tripping of the foot after surgery.

**Figure 2:**
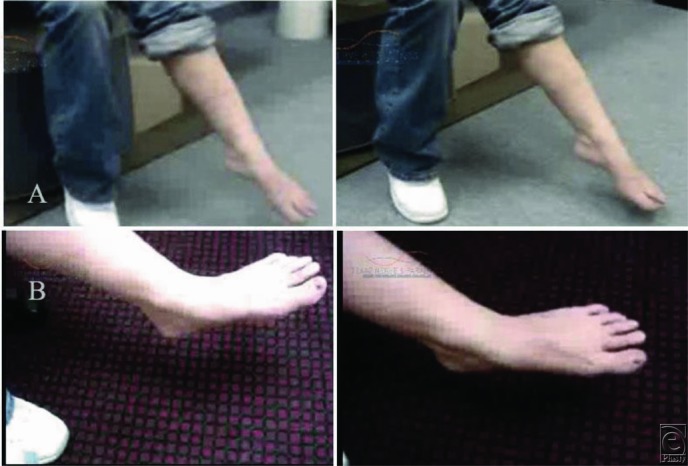

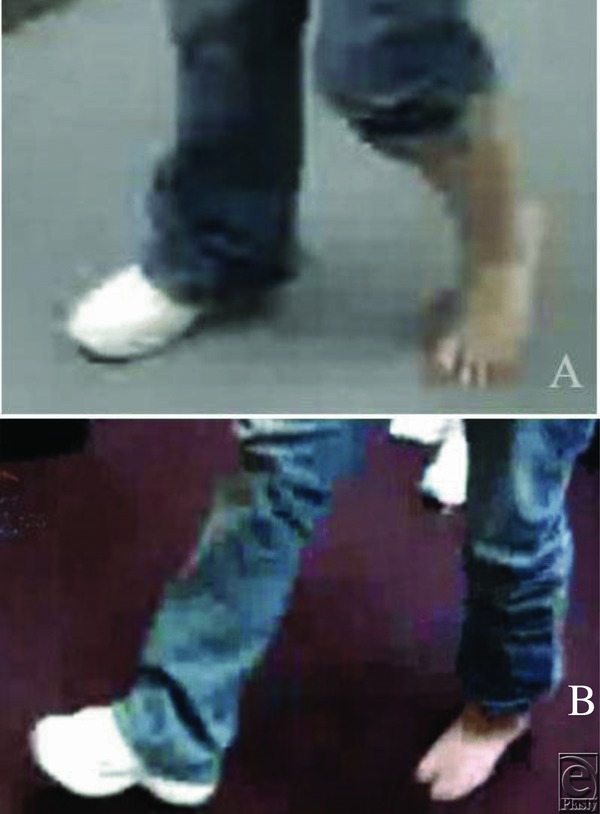
(a) A 26-year-old female patient with left peroneal nerve injury and foot drop resulting from back surgery. Upper panel (A), left foot: No ankle dorsiflexion (BMRC 0/5) and toe extension (BMRC 0/5) before surgery. Lower panel (B): Full recovery of ankle dorsiflexion (BMRC 4+/5) and significant improvement in toe extension after surgery (peroneal nerve decompression, microneurolysis, neuroplasty, and nerve transfer). (b) Upper panel (A): Abnormal walking gait before surgery. Lower panel (B): Improved normal walking gait after surgery.

**Figure 3:**
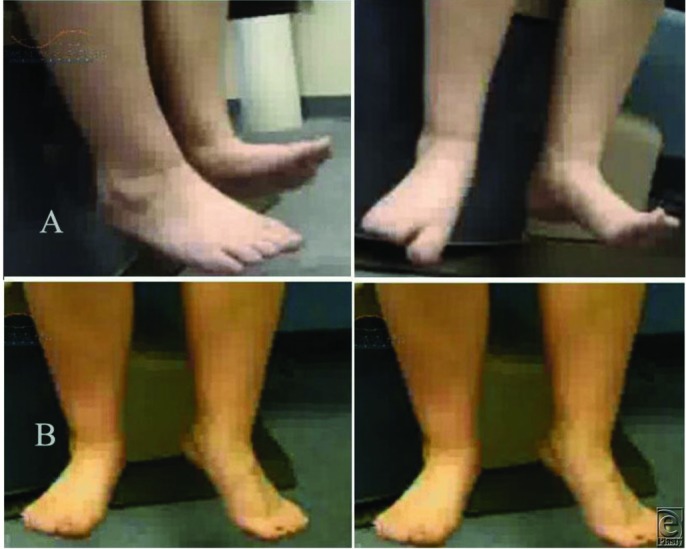

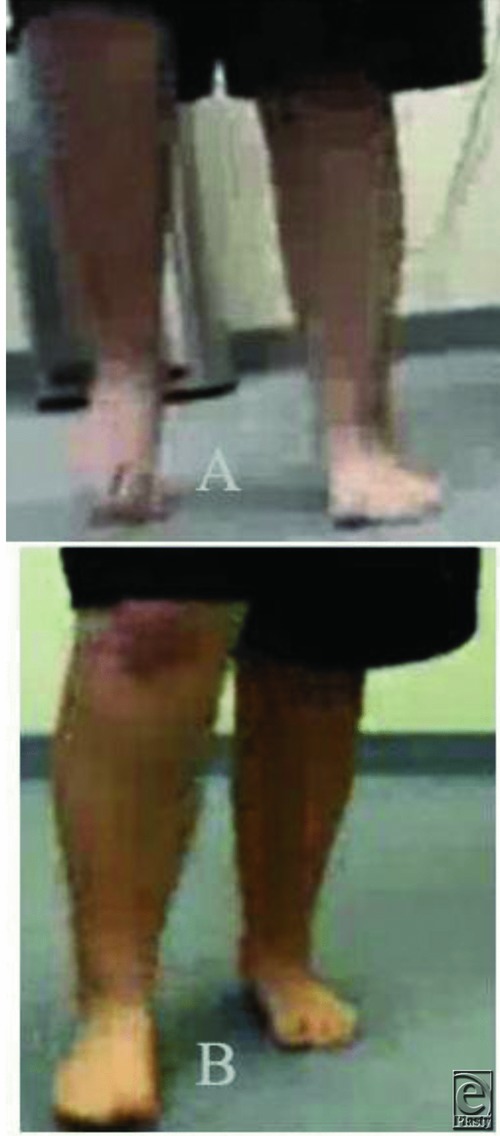
(a) A 16-year-old female patient with right peroneal nerve injury and foot drop resulting from motor vehicle accident. Upper panel (A), right foot: Unable to dorsiflex (BMRC 0/5) the ankle before surgery. Lower panel (B): Gained significant dorsiflexion (BMRC 4/5) after surgery (peroneal nerve decompression, microneurolysis, neuroplasty, and nerve transfer). (b) Upper panel (A), right foot: pathological or neuropathic gait before surgery. Lower panel (B): No steppage gait after surgery.

Of 21 patients who had no ankle dorsiflexion (BMRC 0/5) preoperatively, at least after 6 months of nerve transfer, the outcome was excellent in 9 patients. They had ankle dorsiflexion of BMRC 4/5 and greater (4+/5) and achieved normal gait, indicating that these 9 patients fully recovered anterior tibialis muscle function. In 7 patients, there was significant improvement of dorsiflexion from BMRC 0/5 preoperatively to BMRC 2+ to 3/5 postoperatively and ankle eversion (BMRC 2/5 to 4/5 preoperatively). Some of these patients did not gain complete antigravity, and they were using AFO and braces. Ankle dorsiflexion did not improve in 4 patients. They were considered for tendon transfer (Figs 1–3).

## DISCUSSION

The common peroneal nerve is the most frequently injured nerve in knee dislocation and foot drop, due to lack of intraneural vessels and inconsistent blood supply around fibular neck.^14^ We and other investigators compared and reported the benefits and drawbacks of various foot drop managements.^13^^,^^15^^,^^16^ Giuffre et al^16^ discussed extensively on various treatment strategies and compared specifically our previously published article in nerve transfer in patients with foot drop.

Users of AFOs have been found unsatisfied and noncompliant with the device because of discomfort, hygiene, and mobility issues.^17^^,^^18^ Flores et al^19^ reported that the transfer of the soleus muscle to the deep peroneal nerve demonstrated poor results in most of their patients. Although posterior tibial tendon transfer does not improve ankle dorsiflexion significantly and has long-term complications, it has been considered as a reasonable treatment option.^20^^,^^21^ Nerve grafting is rarely successful when grafts are greater than 6 cm in length.^8^^,^^10^^,^^11^

Peskun et al^22^ found gender (male) as one of the significant risk factors associated with peroneal nerve injury. This is not the case in our present study and might be due to a fewer patients. However, their study did not find that age or mechanism of injury is a predictive risk factor of peroneal nerve injury as shown by Giuffre et al^16^, who showed a trend toward a more favorable outcome in younger patients. Our present study results also do not show any trend of better outcome only in younger patients. In our experience of management of patients with foot drop, older patients, both male and female (52, 53, 54, 57, 64, 77 years of age), also gained ankle dorsiflexion to BMRC 3 and 4 from BMRC 0 after they had nerve transfer procedure. Furthermore, Giuffre et al^16^ compared mechanism of injury (high and low energy) with our previously published report on this subject.^13^ They claim that their patients had high-energy trauma, whereas most of our patients had low-energy injuries.^16^ In addition, they reported that mechanisms of injury have statistically significant effect on the outcome of the surgery.^16^ We disagree with their statements that our patients had low-energy trauma, as all our present study patients had no ankle dorsiflexion (BMRC 0/5) preoperatively. However, overall statistically significant clinical improvement of ankle dorsiflexion and eversion of BMRC 3.6/5 was achieved postoperatively in our present study of patients with foot drop.

Nerve grafts less than 6 cm in length has been shown to achieve antigravity or greater ankle dorsiflexion function in an operative management for common peroneal neuropathy in a series of 318 patients.^23^ Foot movements such as toe extension, ankle dorsiflexion, and ankle eversion (peroneal nerve functions) were very poor (BMRC 1.1/5) preoperatively. Moreover, there was no ankle dorsiflexion (BMRC 0/5) preoperatively in all our patients with foot drop in this study. This indicates that the peroneal nerve injury was severe in our present study patients, unlike Gifurre et al,^16^ stated about the severity of peroneal nerve injury in our patients with foot drop in our previous report.^13^

## CONCLUSION

Overall statistically significant clinical improvement of ankle dorsiflexion, eversion, and toe extension was achieved in 80% of our study patients. Most of these patients gained antigravity and were able to walk with minimal steppage gait. In the other 4 patients (20%), there was good improvement in ankle eversion but poor or no ankle dorsiflexion.

## AUTHOR CONTRIBUTIONS

R.K.N. conceived of the study, performed all the surgical procedures, and revised the manuscript. C.S. participated in the design of the study, gathered data, performed the statistical analysis, and drafted the manuscript. Both authors read and approved the final manuscript.

## Figures and Tables

**Table 1:** Demographics and outcomes of 21 patients with foot drop who had nerve transfer in our present study*

Patient no.	Gender/age at surgery	Cause of Injury	Time to surgery, mo	Donor nerve	Postoperative follow-up, mo	Outcomes
1	M/17	Fell into a ditch and blew out knee in full	2	Tibial	31	Excellent outcome of ankle dorsiflexion and toe extension; partial recovery of eversion
2	F/44	Hip replacement	Unknown	Tibial	16	Excellent outcome of ankle dorsiflexion; partial recovery of eversion
3	M/31	Snowboarding accident	1	Tibial	21	Stable recovery
4	M/48	Lumbar surgery	3	superficial peroneal	6	Good improvement in ankle eversion
5	F/57	Lumbar surgery	15	Superficial peroneal	32	Stable, excellent function of both ankles; no steppage gait
6	F/36	Peroneal ND	2	Tibial	60	Excellent strength grading; gait is much improved with decreased steppage gait
7	F/27	Back surgery hit nerve	4	Superficial peroneal	12	Improvement in walking
8	F/77	Laminectomy	6	Superficial peroneal	-	Active dorsiflexion noted but not yet antigravity
9	F/52	Lumbar disc	5	Tibial	7	Ambulation is with minimal steppage gait
10	F/64	Hematoma	3	Tibial	17	Uses brace only occasionally; continue home Achilles stretches
11	M/24	ACL repair	4	Superficial peroneal	6	Improvement with Gr. 2-2 dorsiflexion and increased eversion; walking normally with AFO
12	M32	Fell off cliff	5	Tibial	14	Continued excellent result
13	M26	Accident	17	Superficial peroneal	16	Increased tone and AROM in dorsiflexion and eversion
14	F59	Back surgery	4		15	Bilateral improvement in right ankle function
15	F53	Post–knee surgery	5	Tibial	18	Steady improvement in function: not using AFO
16	M44	Acetabular repair	10	Superficial peroneal	11	No left ankle dorsiflexion; eversion is significantly improved
17	F16	MVA	1	superficial peroneal	7	Improvement in dorsiflexion
18	F55	Lumbar	4	Superficial peroneal	12	Excellent improvement; walking is also improved
19	F32	Hospitalized due to lupus	4	Superficial peroneal	7	No improvement in dorsiflexion; eversion improved
20	F56	Gunshot	10	Superficial peroneal	18	5/5 strength at ankle; perfect result of nerve transfer surgery
21	M60	Laminectomy	9	Tibial	6	Walking shows minimal steppagge gait; eversion remains good but not dorsiflexion
Mean	44		6		16	Some improvement in ankle dorsiflexion noticed; no AG

*ND indicates nerve damage; ACL, anterior cruciate ligament; AFO, ankle-foot orthosis; AROM, active range of motion; MVA, motor vehicle accident; and AG, anti-gravity.

**Table 2:** Outcomes of peroneal or tibial fascicles transfer in patients with foot drop in our present study

			Preoperative foot movements					Postoperative foot movements				
Patient no.	Gender/age at surgery	Postoperative follow-up, mo	Plantar flexion	Ankle inversion	Ankle dorsiflexion	Ankle eversion	Mean	Plantar flexion	Ankle inversion	Ankle dorsiflexion	Ankle eversion	Mean
1	M/17	31	4+	4+	0	0	2	4+	4+	4	2	3.5
2	F/44	16	5	5	1	3+	3.5	4+	4+	4	2	3.5
3	M/31	21	4+	4+	0	0	2	4+	4+	2	4	3.5
4	M/48	6	4+	4+	0	4+	3	5	5	2+	4	4.0
5	F/57	32	3	3+	0	3	2.3	5	4+	4+	4+	4+
6	F/36	60	4	4	0	3+	2.8	5	5	5	5	5.0
7	F/27	12	4+	4+	0	3+	2.8	4+	4+	4+	2+	3.5
8	F/77	Unknown	4	4	0	1+	2.3	4+	4	2+	4	3.5
9	F/52	7	4+	5	0	3+	3	4+	5	2	4	3.8
10	F/64	17	5	5	0	0	2.5	4+	4+	2	3	3.3
11	M/24	6	4+	4+	0	4	3	5	5	4	4+	4.5
12	M/32	14	4+	4	0	0	2	4+	4	1+	1+	2.5
13	M/26	16	4+	4+	1+	4	3	4+	4+	4+	4+	4.0
14	F/59	15	4+	4+	0	3+	2.8	4+	4+	2+	3+	3.3
15	F/53	18	4+	3+	0	0	1.8	4+	3	0	3	2.5
16	M/44	11	4+	4	0	4	3	4+	4+	1+	4+	3.3
17	F/16	7	4+	4+	0	3+	2.8	4+	4+	4	4+	4.0
18	F/55	12	5	4+	0	2	2.8	4+	4	0	4+	3.0
19	F/52	7	4+	4+	0	4	3	5	5	5	5	5.0
20	M/56	18	4+	4+	0	0	2	4+	4+	0	3	3.3
21	M/60	3	5	4+	0	2	2.8	5	4+	2	3	3.5
Mean	44	16					2.6					3.6
STD							0.5					0.7
*P*										.0000004		
